# Soluble Herpes Virus Entry Mediator and Type II/III Interferons Are Upregulated in Primary Biliary Cholangitis

**DOI:** 10.3390/ijms26020605

**Published:** 2025-01-13

**Authors:** Yooyun Chung, Hio Lam Phoebe Tsou, Michael A. Heneghan, Shilpa Chokshi, Antonio Riva

**Affiliations:** 1 The Roger Williams Institute of Liver Studies, School of Immunology and Microbial Sciences, Faculty of Life Sciences and Medicine, King’s College London & Foundation for Liver Research, London SE5 9NT, UK; 2 King’s College Hospital, London SE5 9RS, UK; 3 Peninsula Medical School, Faculty of Health, University of Plymouth, Plymouth PL4 8AA, UK

**Keywords:** primary biliary cholangitis, cirrhosis, soluble immune checkpoints, bacterial translocation, HVEM, interferon lambda

## Abstract

Bacterial translocation-induced inflammation and immune dysfunction are recognised factors contributing to the pathogenesis of primary biliary cholangitis (PBC). However, the specific involvement of interferons (IFNs) and soluble checkpoints (sol-CRs) in shaping the immune landscape in PBC patients remains unexplored. Furthermore, the influence of ursodeoxycholic acid (UDC) on these immune mediators is unknown. Twenty-eight cytokines and 14 sol-CRs were quantified by Luminex assays in plasma samples from 64 PBC patients and 10 healthy controls (HCs). D-lactate was measured as a marker of bacterial translocation. The PBC subgroups were: 24 UDC responders (UDCRs), 18 UDC non-responders (UDCNRs) and 22 patients with end-stage cirrhotic PBC (ESPBC). Soluble herpes virus entry mediator (HVEM) was upregulated in the UDCR subgroup compared to the HC group (*p* = 0.0404), with increased significance in the ESPBC subgroup (*p* < 0.0001). There was a progressive increase in several sol-CRs, particularly soluble CD80, LAG3 and CD137 in ESPBC patients. IFN-gamma was higher in the ESPBC subgroup compared to the UDCR subgroup. Elevated IFN-gamma in the UDCNR subgroup compared to UDCR was more significant on excluding patients with cirrhosis (*p* = 0.0056). Patients with ESPBC expressed several pro-inflammatory cytokines including IL-6, TNF-alpha and CXCL10 compared to the HC group. IFN-lambda-3, but not IFN-lambda-2, was elevated in the ESPBC subgroup compared to all other subgroups. D-lactate levels were equally elevated in all PBC subgroups compared to the HC group. This study provides valuable insights into the immune landscape of PBC, highlighting potential biomarkers and cytokine signatures associated with disease severity and treatment response. Further investigation into the mechanistic roles may pave the way for more targeted therapeutic interventions in PBC management.

## 1. Introduction

Primary biliary cholangitis (PBC) is a cholestatic autoimmune inflammatory disease of smaller bile ducts characterised by the presence of antimitochondrial antibodies (AMA). Histologically, there is CD4+ and CD8+ T-cell infiltration within the portal tracts and progressive loss of intrahepatic bile ducts with progression to fibrosis and cirrhosis [1,2]. Without treatment, chronic inflammation of the bile ducts progresses to decompensated cirrhosis, the need for liver transplantation (LT), and increased mortality. It has a strong female predominance (10:1 female:male) with peaks in the 5–6th decade [3]. The disease course is heterogeneous and may cause significant symptom burden including pruritus and fatigue.

The first-line therapy, ursodeoxycholic acid (UDC), improves transplant-free survival but up to 40% of patients may have an inadequate biochemical response (BR) [1]. Obeticholic acid (OCA) can be effective in almost 50% of UDC non-responders (UDCNRs) [4]. However, two-thirds of OCA-treated patients experience significant pruritus, requiring antipruritogen medications or discontinuation of therapy [4]. A third of UDCNRs reach BR with bezafibrate, which is used off-licence [5]. Immunosuppression and biologic therapy have not been shown to be effective in PBC [6,7,8,9].

PBC is predominantly mediated by type-1 T helper (Th1) cells and their cytokines. Activated T cells release IFN-γ, TNF-α and IL-4 which, together with macrophage-derived IL-12, stimulate cytotoxic T cells and Th1 cells, with further release of IFN-γ. As the disease progresses, there is a shift from this IL-12/Th1 axis to an IL-23/Th17 profile, characterised by IL-23, IL-6, IL-17 and TGF-β [10,11,12]. PBC patients have higher serum levels of IFN-γ and TGF-β than healthy controls [13,14], but therapeutic targeting of IFN-γ in PBC has not demonstrated significant benefit, hinting at the need for novel approaches [8,15].

Checkpoint receptors (CRs) are integral to maintaining homeostasis of the immunoregulatory pathways [16], and their soluble forms, produced by alternative splicing or ectodomain proteolytic shedding, can potentiate immunoregulatory actions [17,18]. Soluble checkpoint receptors (sol-CRs) are dysregulated in autoimmune diseases and liver diseases related to alcohol and viral hepatitis [18,19,20,21]. Sol-CRs and cytokine expression have been implicated as prognostic tools and have a potential use as targeted immunomodulatory therapy [22,23,24,25]. However, the expression and role of sol-CRs in PBC is not well defined.

The role of the gut–liver axis is being explored in PBC, with findings of reduced diversity, different abundance of several genera, and fewer deconjugated and secondary bile acids (BA) in the stool of untreated PBC patients [26,27]. D-lactate is a by-product of carbohydrate metabolism by intestinal bacteria, and raised plasma levels indicate bacterial translocation from the intestinal lumen to the systemic circulation [18,28,29,30]. Interestingly, changes in the composition of the gut microbiota and intestinal barrier damage, which could raise rates of mucosal bacterial translocation, may be associated with PBC development, progression, prognosis, and response to treatment [31]. A role for type III interferons (IFN-λ) in mucosal and immune modulation has been described in recent years [32,33,34], but their contribution to PBC is unknown.

There is a need for both targeted therapeutic agents with fewer side effects and prognostic biomarkers, which would allow risk stratification and personalised treatment strategies in PBC. The aim of this observational study was to establish the role of sol-CRs, cytokines and bacterial translocation in PBC patients, in association with PBC severity and response to UDC.

## 2. Results

### 2.1. Patient Characteristics

The baseline characteristics of our PBC patients are shown in Table 1. PBC patients were predominantly female (n = 62, 97% female). Age at diagnosis in the UDC responder (UDCR) subgroup was significantly higher than in the UDCNR and end-stage PBC (ESPBC) subgroups (*p* = 0.045). The duration of diagnosis and the age at which the samples were taken for analysis were comparable between PBC subgroups. There was a high prevalence of concomitant autoimmune disease, with the commonest being Sjögren’s or Sicca syndrome, followed by hypothyroidism.

At the time of analysis, two UDCNR patients were on bezafibrate with non-response. After the analysis, two patients received OCA (one achieved BR, one did not respond), four patients received bezafibrate (three achieved BR, one did not respond) and three patients went onto a clinical trial for PBC.

The ESPBC subgroup appeared distinct from the PBC subgroups according to treatment response or disease stage in terms of liver biochemistry and liver prognostic scores. As expected, patients with ESPBC had significantly higher bilirubin, AST, ALP, GGT, IgG and INR compared to UDCRs and UDCNRs but also compared to non-cirrhotic (NC) patients and patients with early cirrhosis (EC). The ESPBC subgroup had lower lymphocyte counts, haemoglobin, platelets and albumin compared to all the other subgroups. This was reflected in the liver prognostic scores, as ESPBC had higher ‘Child–Pugh’ (CP), ‘Model for End-stage Liver Disease’ (MELD), ‘UK model for End-stage Liver Disease’ (UKELD), ‘Aspartate aminotransferase-to-Platelet Ratio Index’ (APRI) and ‘Albumin Bilirubin grade’ (ALBI) scores compared to all subgroups (*p* ≤ 0.0001). Creatinine and total white blood cell counts (WCCs) were progressively lower in NC, EC and (significantly) in ESPBC patients. IgM levels were equally elevated in both EC and ESPBC patients compared to NC. The UDCR and UDCNR subgroups demonstrated no significant difference except for AST, and there was no difference in the prognostic scores. Compared to the HC group, the PBC cohort was older in age (*p* < 0.0001), with a stronger female predominance (*p* = 0.002) and higher white ethnicity (*p* = 0.002).

### 2.2. Soluble Immune Checkpoint Receptors Are Upregulated in Advanced PBC

**Figure 1:**
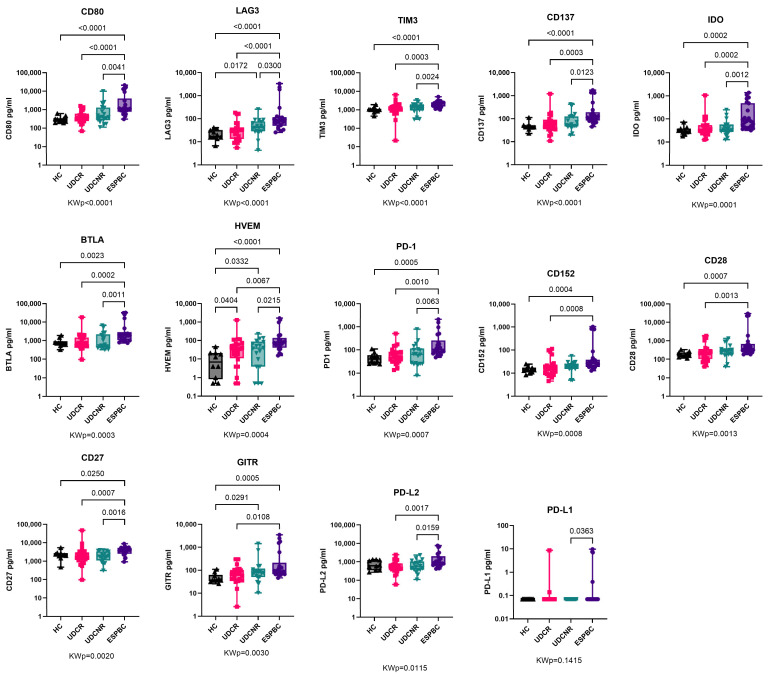
Soluble checkpoint expression in PBC according to treatment response. Soluble HVEM was the only sol-CR to be upregulated in all PBC subgroups compared to HC and the significance increased in ESPBC. Soluble CD80, LAG3, TIM3, and CD137 were the most highly upregulated sol-CRs in EPBC compared to HC. All remaining sol-CRs except for PD-L1 were significantly higher in ESPBC. Soluble PD-L1 was mostly undetectable and similar to controls. KWp, Kruskal–Wallis *p*-value. Boxplots (median, IQR, ±all-value whiskers) ordered by decreasing group-wide statistical significance.

Overall, several sol-CRs progressively increased in concentration between UDCR, UDCNR, and ESPBC, particularly soluble CD80, LAG3, TIM3, CD137, IDO, BTLA, HVEM, and PD-1 (Figure 1, Supplementary Table S1). Soluble HVEM was the only sol-CR to be upregulated in all PBC subgroups compared to the HC group, including UDCR (*p* = 0.0404) and increasingly more in UDCNR (*p* = 0.0332) and ESPBC (*p* < 0.0001) (Figure 1, Supplementary Table S1). Soluble CD80, LAG3, TIM3, and CD137 were the most highly upregulated in the ESPBC subgroup compared to the HC group (*p* < 0.0001, Figure 1, Supplementary Table S1). Of note, no individual sol-CR was different between the UDCR and UDCNR subgroups. In a subanalysis by stage of cirrhosis, levels of several sol-CRs were higher in ESPBC patients compared to either NC or EC, but no differences were observed between NC and EC patients (Supplementary Figure S1, Supplementary Table S1). Soluble PD-L1 was mostly undetectable.

**Table 1:** Clinical features of primary biliary cholangitis patients and controls.

	Healthy Controls (n = 10)	Overall (n = 64)	*p*-Value	UDCR (n = 24)	UDCNR (n = 18)	ESPBC (n = 22)	*p*-Value	Non-Cirrhosis (n = 33)	Early Cirrhosis (n = 9)	ESPBC (n = 22)	*p*-Value
**Age at diagnosis (years)**	---	45 (40.5–52.5)	---	49.5 (44–56.5)	43.5 (38–47)	43.5 (38–48)	**0.045**	47 (41.5–53.5)	44 (38–64.5)	43.5 (38–48)	0.283
**Age at time of sample (years)**	34 (25.8–43.3)	54.5 (48–60.5)	**<0.0001**	57 (48–65.5)	51.5 (50–60)	53.5 (48–58)	0.473	55 (48.5–60.5)	56 (47–68.5)	53.5 (48–58)	0.717
**Sex as self-declared F:M count (% F)**	6:4 (60)	62:2 (97)	**0.002**	23:1 (96)	18:0 (100)	21:1 (95)	0.666	33:0 (100)	8:1 (89)	21:1 (95)	0.212
**Weight (kg)**	---	70 (63–82)	---	75 (67–86.8)	66.5 (63.9–80)	68 (55–77)	**0.048**	73 (64–86)	75 (58–86)	68 (55–77)	0.166
**Ethnicity (counts)**											
** Caucasian**	---	40	**0.002**	10	11	19	**0.028**	17	4	19	**0.021**
** Black**	---	---		---	---	---		---	---	---	
** Asian**	1	2		2	---	---		2	---	---	
** Mixed**	---	---		---	---	---		---	---	---	
** Other**	---	1		---	1	---		---	1	---	
** Unknown**	9	21		12	6	3		14	4	3	
**Autoimmune disease (counts)**											
** Thyroid disease (%)**	---	8 (13)	---	3	2	3	0.308	5	---	3	0.598
** Sjögren’s or Sicca (%)**	---	10 (16)		3	4	3		6	1	3	
** IBD (%)**	---	1 (2)		1	---	---		1	---	---	
** Rheumatoid arthritis (%)**	---	5 (8)		2	3	---		4	1	---	
** Type 1 Diabetes (%)**	---	1 (2)		1	---	---		1	---	---	
** Other (%)**	---	7 (11)		6	1	---		5	2	---	
**WCC (10^9^/L)**	---	5.65 (4.62–6.92)	---	6.09 (5.34–7.30)	6.11 (4.46–7.49)	5.12 (3.37–5.74)	**0.009**	6.39 (5.34–7.68)	4.59 (3.51–6.60)	5.12 (3.37–5.74)	**0.0014**
**Neutrophil (10^9^/L)**	---	3.32 (2.54–4.31)	---	3.58 (2.99–2.49)	3.54 (2.5–4.51)	2.92 (1.84–3.83)	0.131	3.83 (3.07–4.56)	2.70 (2.26–3.69)	2.92 (1.84–3.83)	**0.0183**
**Lymphocyte (10^9^/L)**	---	1.64 (1.14–2.05)	---	1.98 (1.66–2.37)	1.63 (1.27–2.06)	1.21 (0.66–1.62)	**0.0002**	1.85 (1.54–2.25)	1.59 (1.14–2.03)	1.21 (0.66–1.62)	**0.0002**
**Monocyte (10^9^/L)**	---	0.39 (0.31–0.46)	---	0.4 (0.32–0.48)	0.39 (0.30–0.51)	0.37 (0.21–0.42)	0.215	0.41 (0.32–0.48)	0.37 (0.25–0.51)	0.37 (0.21–0.42)	0.162
**Eosinophil (10^9^/L)**	---	0.13 (0.08–0.21)	---	0.12 (0.09–0.19)	0.12 (0.07–0.25)	0.15 (0.08–0.23)	0.465	0.13 (0.08–0.20)	0.11 (0.05–0.25)	0.15 (0.08–0.23)	0.352
**Basophil (10^9^/L)**	---	0.04 (0.02–0.06)	---	0.03 (0.02–0.04)	0.05 (0.03–0.06)	0.03 (0.02–0.05)	0.263	0.04 (0.03–0.06)	0.02 (0.02–0.04)	0.03 (0.02–0.05)	**0.033**
**Haemoglobin (g/L)**	---	128.5 (116.5–136)	---	135 (129–139)	128 (118–139)	117 (109–129)	**0.0007**	133 (128–139)	123 (116–141)	117 (109–129)	**0.0008**
**Platelet (10^9^/L)**	---	194 (131–270.5)	---	216 (182–295)	244 (175–308)	131 (97–180)	**0.0003**	241 (192–309)	121 (80–276)	131 (97–180)	**<0.0001**
**INR**	---	1.03 (0.98–1.12)	---	1.02 (0.99–1.04)	0.96 (0.90–1.00)	1.21 (1.09–1.38)	**<0.0001**	1.00 (0.95–1.03)	1.02 (0.95–1.05)	1.21 (1.09–1.38)	**<0.0001**
**Sodium (mmol/L)**	---	139 (137–141)	---	139.5 (138–141)	139.5 (138–141)	138.5 (136–139)	0.104	140 (138–142)	139 (136–141)	138.5 (136–139)	0.076
**Creatinine (µmol/L)**	---	62.5 (51–72)	---	65 (60–72)	63 (57–76)	49 (46–64)	**0.004**	64 (62–77)	60 (55–69)	49 (46–64)	**0.002**
**Bilirubin (µmol/L)**	---	12 (7–35)	---	7 (6–9)	12 (7–17)	59 (34–94)	**<0.0001**	7 (6–10)	14 (7.5–26)	59 (34–94)	**<0.0001**
**ALT (IU/L)**	---	34 (22–40)	---	23 (18–37)	38 (29–87)	48 (34–61)	**0.032**	29 (22–40)	30 (22–37)	48 (34–61)	0.648
**AST (IU/L)**	---	49 (30–101)	---	28 (21–35)	55 (35–92)	112 (68–156)	**<0.0001**	34 (26–49)	45 (27–103)	112 (68–156)	**<0.0001**
**ALP (IU/L)**	---	194 (124–348)	---	122 (104–139)	283 (198–390)	348 (175–530)	**<0.0001**	142 (116–237)	190 (125–532)	348 (175–530)	**0.0008**
**GGT (IU/L)**	---	123 (45–375)	---	36 (19–70)	259 (216–517)	307 (94–480)	**<0.0001**	65 (23–238)	211 (82–243)	307 (94–480)	**0.011**
**Albumin (g/L)**	---	41 (36–45)	---	44 (41–46)	43 (40–45)	34 (32–38)	**<0.0001**	44 (42–46)	41 (38–45)	34 (32–38)	**<0.0001**
**IgM (g/L)**	---	3.57 (2.56–4.79)	---	2.35 (1.3–3.64)	3.05 (2.28–4.61)	4.01 (2.69–6.42)	0.131	2.78 (1.60–3.45)	5.53 (3.42–8.05)	4.01 (2.69–6.42)	**0.007**
**IgG (g/L)**	---	16.97 (12.71–22.66)	---	14.63 (11.87–18.09)	12.9 (11.47–13.31)	21.53 (17.14–24.49)	**0.001**	12.59 (11.30–14.35)	13.68 (13.09–17.13)	21.53 (17.14–24.49)	**0.0005**
**Protein (g/L)**	---	76 (72–79)	---	76 (73–79)	76.5 (71–80)	74.5 (71–78)	0.884	76 (73–80)	72 (69–81)	74.5 (71–78)	0.596
**Child–Pugh score**	---	5 (5–7)	---	5 (5–5)	5 (5–5)	8 (7–8)	**<0.0001**	-	5 (5–5)	8 (7–8)	**<0.0001**
**MELD**	---	7 (6–13)	---	7 (6–7)	6 (5–7)	13 (11–15)	**<0.0001**	6 (6–7)	7 (7–8)	13 (11–15)	**<0.0001**
**UKELD**	---	47 (45–51)	---	45 (43–47)	46 (44–48)	53 (51–54)	**<0.0001**	45 (43–47)	47 (45–49)	53 (51–54)	**<0.0001**
**ALBI**	---	−2.84 (−3.20–−1.89)	---	−3.18 (−3.34–−3.00)	−3.09 (−3.20–−2.65)	−1.67 (−1.91–−1.55)	**<0.0001**	−3.12 (−3.35–−3.02)	−2.65 (−3.29–−2.36)	−1.67 (−1.91–−1.55)	**<0.0001**
**APRI**	---	0.55 (0.28–1.41)	---	0.27 (0.17–0.39)	0.43 (0.28–0.89)	1.72 (1.16–2.32)	**<0.0001**	0.28 (0.20–0.42)	0.69 (0.32–1.89)	1.72 (1.16–2.32)	**<0.0001**
**UDCA (mg/kg)**	---	13 (11–15)	---	12 (10–13)	13 (12–15)	14 (11–16)	0.148	12 (11–14)	13 (11–16)	14 (11–16)	0.31
**Time from diagnosis to sample (years)**	---	8 (4–13)	---	5 (3–11)	9 (6–13)	10 (4–13)	0.188	6 (4–12)	9 (4–13)	10 (4–13)	0.558

ALBI = albumin bilirubin grade, ALP = alkaline phosphatase, ALT = Alanine aminotransferase, APRI = Aspartate aminotransferase-to-platelet ratio index, AST = Aspartate aminotransferase, F = female, GGT = gamma-glutamyl transferase, IBD = inflammatory bowel disease, Ig = immunoglobulin, INR = international normalised ratio, M = male, MELD = Model for end-stage liver disease, UDCA = ursodeoxycholic acid, UKELD = United Kingdom Model for End-Stage liver disease, WCC = white cell count. Unless indicated differently, all the values are represented as “median (Q1–Q3)”.

### 2.3. Pro-Inflammatory Cytokines Are Upregulated in End-Stage PBC

**Figure 2:**
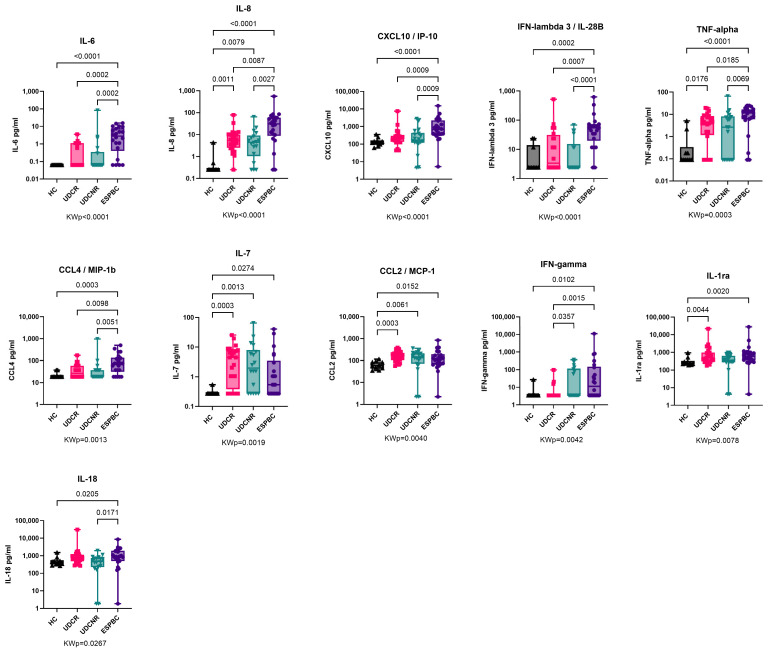
Cytokine expression in PBC according to treatment response. Cytokine expression of IL-6, CXCL10, TNF-α, IFN-λ3, and IFN-γ increased as disease progressed to ESPBC. IL-8, IL-7, and CCL2 were elevated in PBC subgroups compared to the HC group. The following cytokines were comparable across groups and are not included in this figure: IL-23, IL-2, Osteopontin, IL-33, IFN-α, CCX3/MIP-1α, IFN-λ2, IL-17A, IL-15, IL-12p70, IL-4, GM-CSF, IL-13, IL-1α, IL-5, IL-1β, and IL-10. KWp, Kruskal–Wallis *p*-value. Boxplots (median, IQR, ±all-value whiskers) ordered by decreasing group-wide statistical significance.

The expression of several predominantly pro-inflammatory cytokines/chemokines increased as the disease phenotype changed from UDCR to ESPBC, including IL-6, CXCL10, IFN-λ3, TNF-α, and IFN-γ (Figure 2, Supplementary Table S1). All PBC subgroups according to treatment response had upregulation of CCL2, IL-7, and IL-8 compared to the HC group. Levels of CCL2 and IL-7 were significantly higher in the UDCR subgroup compared to the HC group (*p* = 0.0003 for both), and this difference was maintained in the UDCNR and ESPBC subgroups. Interestingly, IFN-λ3, but not IFN-λ2, was elevated in ESPBC patients when compared to the other subgroups, and IFN-λ2 was mostly undetectable in PBC patients and HCs. In a subanalysis by cirrhosis stage, no differences in cytokine levels were observed between NC and EC patients (Supplementary Table S1).

**Figure 3:**
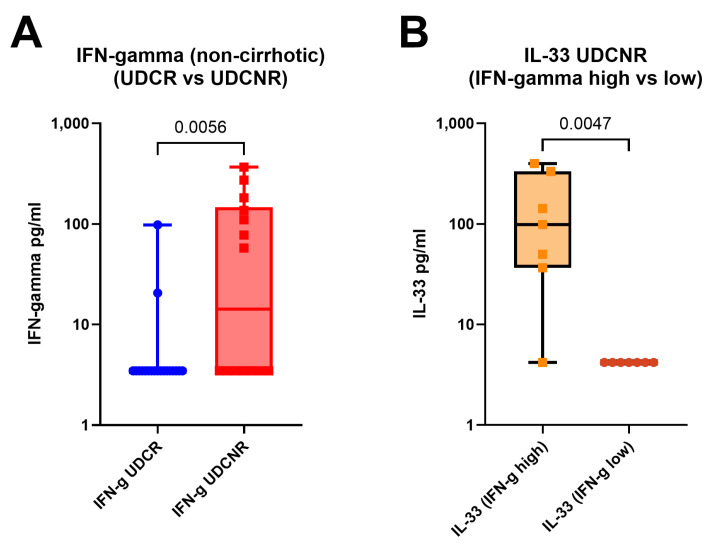
IFN-γ levels remain elevated in the UDCNR subgroup compared to the UDCR subgroup on exclusion of early cirrhosis (**A**). IFN-γ cluster analysis within UDCNR showed that the high IFN-γ cluster was associated with increased IL-33 (**B**). Mann–Whitney *p*-values. Boxplots (median, IQR, ±all-value whiskers).

IFN-γ levels progressively increased as the disease phenotype changed from UDCR to UDCNR (*p* = 0.0357) and ESPBC (*p* = 0.0015). In the ESPBC subgroup, elevated IFN-γ was accompanied by increased CXCL10. IFN-γ levels were comparable in the UDCR and HC groups, and IFN-γ was the only cytokine differentiating the UDCR subgroup from the UDCNR subgroup (*p* = 0.0357). This signature was maintained also when considering only non-cirrhotic/NC patients, suggesting a cirrhosis-independent role of IFN-γ in influencing UDC response (MWp = 0.0056, Figure 3A). Amongst NC patients, most in the UDCR subgroup had low or undetectable levels of IFN-γ but, surprisingly, IFN-γ levels could discriminate two clear subsets of UDCNR patients: half (n = 7) with highly detectable IFN-γ and half with undetectable levels. When we investigated whether other cytokines were different between IFN-γ high and IFN-γ low NC UDCNR patients, we found that IFN-γ-high patients also had detectable IL-33, whereas IL-33 was undetectable in the IFN-γ-low subset (MWp = 0.0047, Figure 3B). No clinical features were associated with this peculiar difference in IFN-γ expression amongst NC UDCNR patients.

### 2.4. Bacterial Translocation Is Increased in Primary Biliary Cholangitis

**Figure 4:**
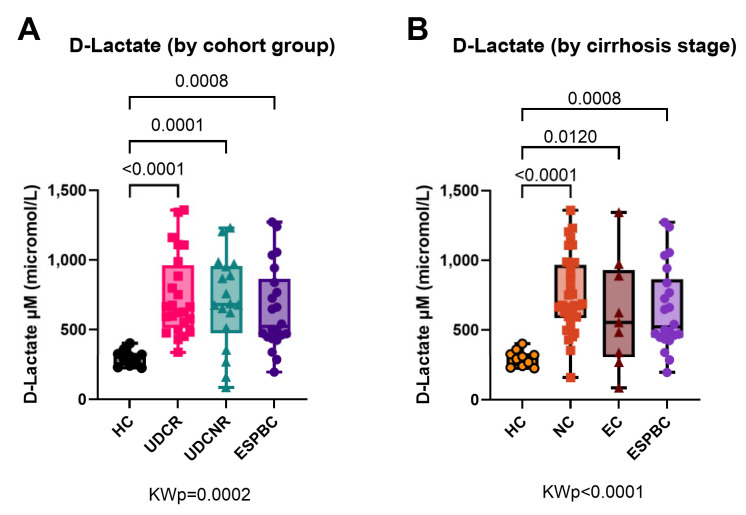
D-lactate as a surrogate marker of bacterial translocation in PBC according to treatment response (**A**) or stage of cirrhosis (**B**). All PBC subgroups had equally high levels compared to the HC group. KWp, Kruskal–Wallis *p*-value; Boxplots (median, IQR, ±all-value whiskers).

D-lactate levels were equally elevated in all PBC subgroups when compared to the HC group regardless of type of UDC response (KWp = 0.0002, Figure 4A) or stage of cirrhosis (KWp < 0.0001, Figure 4B). In a subanalysis by cirrhosis stage, D-lactate levels were comparably high in the UDCR and UDCNR subgroups in both NC and EC patients (Supplementary Figure S2). When checking for bowel pathology amongst the PBC patients, no patients were found to have active bowel disease at the time of analysis, and only two patients had had a partial colectomy more than 10 years prior.

### 2.5. Soluble Checkpoint Receptors and Cytokines Correlate with Liver Prognostic Scores

**Figure 5:**
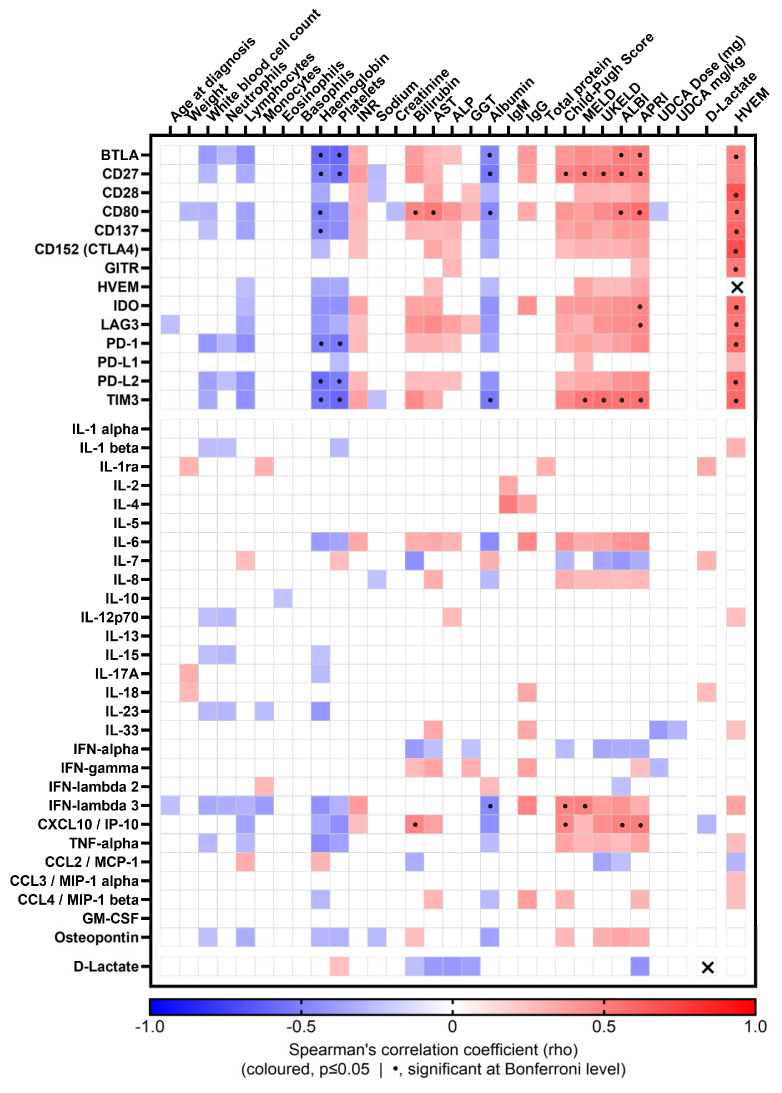
Correlation matrix of soluble CRs and cytokines with clinical parameters and D-lactate in PBC patients. Coloured matrix squares correspond to significant Spearman’s correlation coefficients (*p* ≤ 0.05); the colour scale represents the direction and strength of the correlation; blank squares indicate lack of significant correlation; dotted squares indicate correlations significant at Bonferroni level; crossed squares indicate same-molecule correlations, therefore not considered (HVEM:HVEM, rho = 1; D-Lactate:D-Lactate, rho = 1).

Soluble TIM3 and CD27 showed strong positive correlation with the majority of liver prognostic scores (Figure 5) including the MELD, UKELD, ALBI and APRI scores; CD27 correlated also with the CP score. This was reflected in the biochemistry, as both sol-CRs demonstrated negative correlations with haemoglobin, platelet count and albumin. Soluble BTLA and CD80 positively correlated with the ALBI and APRI scores.

The pro-inflammatory cytokine IFN-λ3 showed positive correlations with the CP and MELD scores, whereas CXCL10 positively correlated with the CP in addition to the ALBI and APRI scores (Figure 5).

Soluble HVEM was positively correlated with several other soluble immune checkpoints, whereas D-lactate was only weakly correlated with few clinical and immune parameters (Figure 5).

### 2.6. HVEM Gene Expression Is Upregulated in Immune Cells from PBC Patients

**Figure 6:**
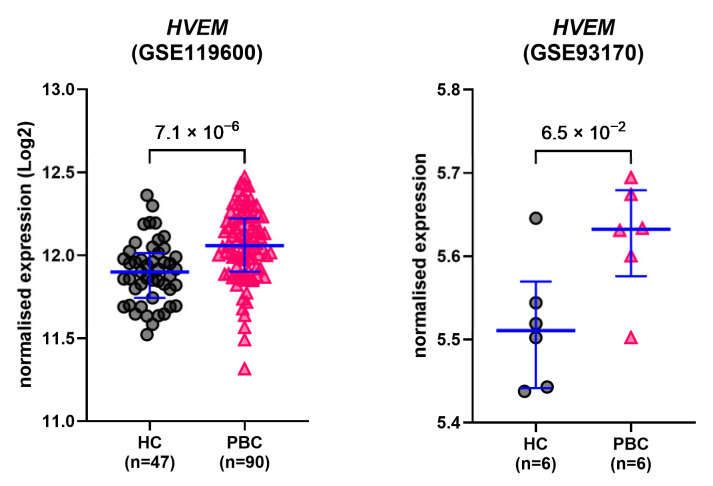
GEO datasets. Gene expression levels of HVEM in PBC patients and healthy controls. Data were extracted from two publicly available GEO datasets (GSE119600 [35] and GSE93170 [36]) and analysed for differential expression. Lines and error bars: median and 1st–3rd quartiles. *p*-values: Mann–Whitney U test.

Differential expression analysis of data extracted from publicly available GEO datasets GSE119600 [35] and GSE93170 [36] showed that the expression of *HVEM* was elevated in PBC patients compared to HCs in both whole-blood transcriptome (GSE119600, MWp = 7.1 × 10^−6^; HC/PBC n = 47/90) and (to a lesser extent) in CD4+ T cells (GSE93170, MWp = 0.065; HC/PBC n = 6/6) (Figure 6). No significant difference in *IFNL1*, *IFNL2* or *IFNL3* expression was detected between the two groups in the two datasets.

## 3. Discussion

PBC has an unpredictable treatment response with limited therapeutic agents and prognostic markers. In this study, we discovered that, amongst a general increase in cytokines and sol-CR expression in PBC patients (primarily ESPBC), the type II/III interferon system and soluble HVEM may play important roles in disease progression and response to UDC treatment. IFN-γ was the only cytokine able to discriminate UDC responders and non-responders independently of cirrhosis, and ESPBC patients displayed a selective upregulation of IFN-λ3, as IFN-λ2 was instead mostly undetectable in all groups. Furthermore, soluble HVEM was the only sol-CR comparably raised in all PBC patients, including a significant upregulation in the UDCR subgroup compared to the HC group, thus attaining a role as biomarker of PBC. Finally, we found that PBC is also characterised by increased bacterial translocation, independently of cirrhosis and of response to UDC. While IFN-γ appears to be a possible marker of treatment response and IFN-λ3 selectively identifies severe disease stages, soluble HVEM and bacterial translocation would thus appear to be features of all stages of PBC.

IFN-γ displayed a stepwise increase as the disease phenotype changed from UDCR to UDCNR and this difference was maintained on exclusion of patients with (early) cirrhosis. Increase in IFN-γ was accompanied by elevated chemokines CCL2 and CXCL10, possibly suggesting mobilisation of monocyte/macrophages and T cells. Furthermore, amongst patients in the UDCNR subgroup, IFN-γ appeared to differentiate two subgroups of patients, also featuring differential levels of IL-33 but no identifiable clinical differences in parameters collected as part of routine patient care. In the Mdr2−/− murine model of cholestasis, there was increased IFN-γ, CXCL10, and hepatic macrophages with elevated CCL2 [37]. IFN-γ was mainly derived from CD8+ T cells and NK cells, and depletion of T cells or NK cells reduced IFN-γ, which resulted in improved hepatic inflammation and reduced CD8+ T-cell cytotoxicity, hepatic fibrosis, and CCL2 levels. Moreover, treatment with anti-IFN-γ antibodies resulted in improved fibrosis. All IFNs rely on the JAK-STAT pathway and a genome-wide association study identified the JAK-STAT pathway as a therapeutic target for PBC [38]. Future trials using a JAK1/2 inhibitor may be more beneficial in earlier stages of PBC before cirrhosis and assessment of treatment response using markers of inflammation. The identification of two subsets of UDC non-responders, based on IFN-γ detectability, is interesting per se and although we did not find any clinical correlations it would be interesting to perform longitudinal studies to investigate whether this feature may be a prognostic factor for progression or differential types of treatment responses in the longer term. The link between IFN-γ and IL-33 in UDCNR patients is also of potential mechanistic interest, as IL-33 may be linked to local tissue-related inflammation and may be important for intestinal/mucosal health, also playing a role in type II inflammatory antiparasitic responses. Future studies should investigate and clarify the link between IFN-γ and IL-33 as mechanistic and prognostic biomarkers in PBC.

In this study, IFN-λ3 was upregulated as the disease phenotype changed from UDCR to ESPBC whereas IFN-λ2 levels remained comparable to those in the HC group. IFN-λ subtypes 1–4 form the type III IFNs, whose receptors are predominantly expressed in hepatocytes, barrier epithelial cells and immune cells, such as dendritic cells, NK cells and B cells [34]. Type III IFNs have similar antiviral activity to type I IFNs, though type I receptors are ubiquitously expressed. IFN-λ3 has been implicated in autoimmune and inflammatory conditions [39,40]. In a study of 93 lupus patients, serum IFN-λ3, but not IFN-λ1 or 2, was significantly higher in patients compared with controls. Serum IFN-λ3 correlated with the severity of lupus activity, particularly with active serosal and cutaneous disease [40]. In a similar way, our data demonstrated that IFN-λ3, but not IFN-λ2, is increasingly expressed in ESPBC.

Single nucleotide polymorphisms (SNPs) in the IFN-λ3/4 genes have been identified as risk factors for development of lupus, hepatic inflammation and subsequent liver fibrosis in both viral hepatitis and non-alcohol-related fatty liver disease [41,42,43,44,45]. Investigation of IFN-λ3/4 SNPs within PBC may identify potential biomarkers for susceptibility to PBC. IFN-λ3 levels were recently found to prognosticate patients with COVID-19 infection [46] and our data would support the evaluation of IFN-λ3 as a prognostic tool in PBC.

Moreover, therapeutic applications of IFN-λ3 may be appealing due to the selective expression of its receptors, which makes type III IFNs possibly less prone to systemic side effects compared to type I IFNs. IFN-λ3 has been engineered to increase the affinity for its receptor and enhance its activity in vivo [47]. The selective increase in IFN-λ3 in PBC would support future exploration of IFN-λ3 as a potential therapeutic target.

A novel finding of this study was that soluble HVEM is the only sol-CR to be upregulated in UDCRs compared to HCs and this difference is also maintained at later stages of disease, making soluble HVEM a PBC-wide biomarker. Soluble HVEM has been investigated in liver disease of other aetiologies, including alcohol-related liver disease (ArLD) and hepatitis B, but with mixed results [18,19,20]. In ArLD, soluble HVEM was downregulated compared to levels in HCs [18,20], although another publication found its levels to be higher in ArLD patients and linked to immune regulation [21].

HVEM is a member of the tumour necrosis factor (TNF) receptor superfamily and has a role in herpes virus entry, T-cell activity and tumour immunity [48]. It is expressed in several immune cells including CD4+ and CD8+ T cells, B cells, dendritic cells, and NK cells, as well as in the liver, lungs, and intestinal epithelial cells [49]. HVEM has multiple binding sites and ligands, including BTLA (co-inhibitory), CD160, and LIGHT (co-stimulatory), whilst also having bidirectional activity [50]. Soluble HVEM is thought to be produced through shedding of the ectodomain and its role is being explored in cancer immunotherapy [51].

Both soluble and membrane HVEM bind to CD160, which is highly expressed on NK cells, promoting cytotoxicity and inflammatory cytokine production [52]. Our data also showed increased expression of several pro-inflammatory cytokines in ESPBC including TNF-α, IL-6, and IFN-γ, although causal links between soluble HVEM and cytokine expression could not be investigated in our study due to sample availability.

In keeping with our study, soluble HVEM levels are elevated in other autoimmune inflammatory conditions such as rheumatoid arthritis, psoriasis, and Behçet’s disease [49]. HVEM has been implicated in inflammatory bowel disease, where TNF is a key pro-inflammatory cytokine. Soluble HVEM levels are increasingly upregulated in advanced stages of breast, gastric, and hepatocellular carcinomas indicating a potential use of HVEM as a prognostic marker [23,51,53]. In our data, soluble HVEM was the only sol-CR to be elevated in the UDCR subgroup compared to the HC group and may have a role as a prognostic tool in PBC.

In a recent study, soluble CD134, LAG3, PD-1, PD-L1, and TIM3 became increasingly elevated in advanced stages (as defined by histological criteria) of untreated PBC patients [54]. Soluble PD-L1 was almost undetectable in our PBC patients, in keeping with the previous histology findings of membranous PD-L1 [13,55]. The patients in our data were all treated with UDC at the time of assessment, which may account for this difference. Another study focusing on autoimmune hepatitis (AIH)/PBC variants found that the levels of expression of soluble LAG3, TIM3, CD86, and CD25 in AIH were higher and could be differentiated from PBC [56]. Neither study quantified soluble HVEM. In our data, soluble LAG3 and TIM3 were elevated in ESPBC, though we do not have AIH patients for comparison. Soluble TIM3 has been implicated in ArLD as well as AIH [18,20]. Elevation in soluble TIM3 may be less specific to the aetiology and reflect the severity of liver disease, which is in keeping with our data as soluble TIM3 correlated with the liver prognostic scores.

D-lactate was equally elevated in PBC subgroups, indicating increased bacterial translocation in all PBC patients. Comparably high values between patient subgroups were also accountable for the apparent lack of clinical and immunological correlations involving this bacterial translocation marker. There is increased gut permeability in PBC patients compared to non-PBC liver disease, as demonstrated by oral sucrose and lactulose-mannitol absorption in the gastroduodenum and intestine, respectively [57], possibly linked to IFN-γ–associated effects on the intestinal epithelium [58,59,60]. Elevated bile acids in a cholestatic mouse model were shown to upregulate hepatic macrophage inflammasomes, which increased intestinal permeability [61]. This may have implications for the development of liver disease. Repeated inoculation of BALB/c mice with bacteria led to portal inflammation and biliary epithelial damage, followed by portal tract fibrosis [62]. PBC patients have been reported to have higher serum levels of gut endotoxins/lipopolysaccharide, with increased expression of toll-like receptor (TLR) 4 and pro-inflammatory cytokines TNF-α, IL-1β, IL-6, and IL-8 compared to non-PBC liver disease [10].

Targeting the gut–liver axis in cholestasis has been explored in animal models. Treatment with antibiotics in a cholestatic mouse model led to reduced CD8+ and CD4+ T cells and colony-forming units in the liver [63]. The probiotic *Lactobacillus rhamnosus GG* (LGG) in cholestatic mice resulted in improved liver biochemistry and fibrosis via increased FXR–FGF-15 signalling to suppress bile acid synthesis, as well as enriching the gut microbiota with bacteria containing bile salt hydrolase [64]. Our data would support the modulation of the gut–liver axis as a therapeutic target in PBC.

To validate the findings of this study, data on *HVEM* and *IFNL* genes from public GEO datasets were analysed for differential expression between HCs and PBC patients. Both datasets showed upregulation of *HVEM* in PBC patients compared to HCs, which is consistent with our findings and strengthens the hypothesis that HVEM may play a role in PBC immunopathogenesis. A possible limitation lies in the need to clarify the relationship between gene transcription and production of soluble forms of immune checkpoints [65]. Current kits for the measurement of soluble immune checkpoints are unable to answer this question, and further molecular studies are therefore warranted, particularly in light of published evidence demonstrating a possible biological role for soluble HVEM [21].

Neither dataset showed differences in *IFNL* gene expression, which contrasts with our findings that IFN-λ3 was elevated in the ESPBC group when compared to other subgroups. The biology of the IFN-λs is complicated by the interplay between several genetic polymorphisms modulating gene expression and the relative balance between different IFN-λ types [44,66]. Open questions remain in relation to the possible hepatic or immune source of these cytokines, and published evidence points to the intestinal mucosa as a possible target organ for this class of cytokines [32,33,67]. The link with mucosal health is consistent with our data showing increased bacterial translocation in PBC patients, compared to healthy controls, which strengthens the previously observed association with cirrhosis in other aetiologies [18,29,30,68].

The limitations of this study include the cross-sectional design, which does not shed light on the causal mechanisms of sol-CR and cytokines in PBC. However, the subgroups according to treatment response and stage of PBC can give some indications to which sol-CR and cytokines may be involved in the different treatment response or stages of the disease. To explore this further, treatment-naïve cohorts, longitudinal follow-ups and in vitro immune cell investigations, to analyse the changes in sol-CR and cytokine expression and consequent immunomodulation, would provide valuable insight.

To conclude, IFN-γ levels increased as disease progressed and discriminated UDC responders and non-responders independently of cirrhosis, whereas IFN-λ3 may have a role in advanced PBC that is distinct to IFN-λ2. Soluble HVEM and bacterial translocation were elevated in all PBC subgroups and increasingly upregulated as disease progressed. These may serve as potential biomarkers and/or as novel therapeutic targets in PBC and warrant further exploration.

## 4. Materials and Methods

### 4.1. Patients

Written informed consent was obtained from all patients in the study and the study protocol conforms to the ethical guidelines of the Declaration of Helsinki (IRAS 244089, REC 19/NW/0750). Plasma samples from 70 PBC patients recruited between 2012–2022 at King’s College Hospital were compared to 10 healthy controls (HCs). PBC diagnosis was based on the European Association for the Study of the Liver (EASL) guidelines. All patients in our cohort had positive AMA M2 subtype (+/− ANA) or liver biopsy consistent with PBC. Liver disease of other aetiology and overlap syndromes with autoimmune hepatitis (AIH) were excluded.

Clinical data on demographics, medications, comorbidities, duration of diagnosis, autoantibody profiles, liver biochemistry, and presence of cirrhosis were extracted from electronic medical records. Liver prognostic scores Child–Pugh (CP), Model for End-stage liver disease (MELD), United Kingdom Model for End-Stage liver disease (UKELD), albumin bilirubin grade (ALBI) and aspartate aminotransferase-to-platelet ratio index (APRI) at time of the plasma sample collection were noted.

The PBC subgroups were classified according to Paris I criteria of treatment response and stage of disease. The clinical response groups were UDC responder (UDCR, n = 24), UDC non-responder (UDCNR, n = 18) and end-stage PBC prior to liver transplantation (ESPBC, n = 22). Smaller subgroups included UDC intolerant (n = 4) and latent PBC (n = 2), which were excluded from the analysis due to small subgroup size. According to the stage of disease, there were 33 non-cirrhotic (NC) PBC patients (19 UDCR, 14 UDCNR); 9 early cirrhosis (EC) with Child–Pugh score A (5 UDCR, 4 UDCNR); and 22 advanced cirrhosis with ESPBC. All baseline demographic and clinical characteristics are summarised in Table 1.

### 4.2. Plasma Isolation

Plasma samples were isolated from EDTA-anticoagulated whole blood by centrifugation at 939× *g* for 15 min and cryopreserved at −80 °C for subsequent analyses.

### 4.3. Luminex Assays for Soluble Immune Checkpoints and Cytokines

Luminex multiplex ELISAs were conducted to measure plasma levels of sol-CRs and cytokines. The Immuno-Oncology Checkpoint 14-plex Human Procartaplex panel 1 (Thermo Fisher Scientific, Altrincham, UK) was used to measure sol-CRs BTLA, CD27, CD28, CD80, CD137 (4-1BB), CD152 (CTLA4), GITR, HVEM, IDO, LAG3, PD-1, PD-L1, PD-L2, and TIM3. A human Luminex 28-Plex Discovery Assay (Bio-Techne/R&D systems, Abingdon-Oxford, United Kingdom) was used to measure the following cytokines: IL-1α, IL-1β, IL-1ra, IL-2, IL-4, IL-5, IL-6, IL-7, IL-8, IL-10, IL-12p70, IL-13, IL-15, IL-17A, IL-18, IL-23, IL-33, IFN-α, IFN-γ, IFN-λ2 (IL-28A), IFN-λ3 (IL-28B), CCL2/MCP-1, CCL3/MIP-1α, CCL4/MIP-1β, CXCL10/IP-10, GM-CSF, Osteopontin, and TNF-α. All the assays were performed according to manufacturer’s instructions without any protocol modifications. All the quantifications were evaluated with a MAGPix instrument with xPonent v4.2 software (LuminexCorp, Hertogenbosch, The Netherlands).

### 4.4. Plasma D-Lactate Quantification

Plasma D-lactate was quantified as a surrogate marker of bacterial translocation using a colorimetric assay kit, following manufacturer’s instructions without any protocol modifications (AbCam, Cambridge, UK).

### 4.5. GEO Datasets

Two publicly available GEO datasets, GSE119600 [35] and GSE93170 [36], were accessed and data on HVEM (*TNFRSF14*, also known as *HVEM*) and interferon lambda (*IFNL1*, *IFNL2*, *IFNL3*) genes were extracted for differential expression analysis between healthy controls and PBC in whole-blood transcriptome and CD4+ T cells, respectively.

### 4.6. Statistical Analysis

Group comparisons for continuous variables (including gene expression data from the publicly available GEO datasets) were performed using the Mann–Whitney (MW) U test or the Kruskal–Wallis (KW) test as appropriate. Dunn’s test was used for multiple comparisons following a significant KW result, considering each comparison as stand-alone. Comparisons for categorical variables were performed using the Chi-squared test or Fisher’s exact test. Correlations were investigated by Spearman’s correlation analysis, with Bonferroni correction for test multiplicity. Due to the strong sex dimorphism (‘sex’ as assigned at birth) specific to PBC, it was not possible to perform sex-based comparisons or analyses disaggregated by sex. Statistical significance was set at alpha = 0.05. Statistical analyses were performed using IBM SPSS v28 (Armonk, NY, USA), GraphPad Prism 9 (San Diego, CA, USA) and Microsoft Excel 365 (Redmond, WA, USA). Unless stated differently, data are presented as median with interquartile range (Q1, Q3).

## 5. Conclusions

Amongst several soluble checkpoint receptors and pro-inflammatory cytokines that were found to be elevated in patients with advanced/end-stage PBC (including soluble CD80, soluble LAG3, soluble CD137, IL-6, TNF-α, and CXCL10), our key findings highlight that IFN-γ levels increased as disease progressed and discriminated UDC responders and non-responders independently of cirrhosis, whereas elevated IFN-λ3 may have a role in advanced PBC that is distinct from IFN-λ2. Soluble HVEM and bacterial translocation were elevated in all PBC subgroups and increasingly upregulated as disease progressed.

This study provides valuable insights into the immune landscape of PBC, highlighting potential biomarkers and cytokine signatures associated with disease severity and treatment response. These may serve as novel therapeutic targets in PBC and warrant further exploration for more targeted interventions in PBC management.

## Data Availability

Data may be made available upon reasonable written request to the corresponding author.

## Supplementary Materials

The following supporting information can be downloaded at: https://www.mdpi.com/article/10.3390/ijms26020605/s1.

## References

[1] (2017). EASL Clinical Practice Guidelines: The diagnosis and management of patients with primary biliary cholangitis. J. Hepatol..

[2] Lindor K.D., Bowlus C.L., Boyer J., Levy C., Mayo M. (2019). Primary Biliary Cholangitis: 2018 Practice Guidance from the American Association for the Study of Liver Diseases. Hepatology.

[3] Griffiths L., Dyson J.K., Jones D.E. (2014). The new epidemiology of primary biliary cirrhosis. Semin. Liver Dis..

[4] Nevens F., Andreone P., Mazzella G., Strasser S.I., Bowlus C., Invernizzi P., Drenth J.P., Pockros P.J., Regula J., Beuers U. (2016). A Placebo-Controlled Trial of Obeticholic Acid in Primary Biliary Cholangitis. N. Engl. J. Med..

[5] Corpechot C., Chazouillères O., Rousseau A., Le Gruyer A., Habersetzer F., Mathurin P., Goria O., Potier P., Minello A., Silvain C. (2018). A Placebo-Controlled Trial of Bezafibrate in Primary Biliary Cholangitis. N. Engl. J. Med..

[6] Tsuda M., Moritoki Y., Lian Z.X., Zhang W., Yoshida K., Wakabayashi K., Yang G.X., Nakatani T., Vierling J., Lindor K. (2012). Biochemical and immunologic effects of rituximab in patients with primary biliary cirrhosis and an incomplete response to ursodeoxycholic acid. Hepatology.

[7] Hirschfield G.M., Gershwin M.E., Strauss R., Mayo M.J., Levy C., Zou B., Johanns J., Nnane I.P., Dasgupta B., Li K. (2016). Ustekinumab for patients with primary biliary cholangitis who have an inadequate response to ursodeoxycholic acid: A proof-of-concept study. Hepatology.

[8] de Graaf K.L., Lapeyre G., Guilhot F., Ferlin W., Curbishley S.M., Carbone M., Richardson P., Moreea S., McCune C.A., Ryder S.D. (2018). NI-0801, an anti-chemokine (C-X-C motif) ligand 10 antibody, in patients with primary biliary cholangitis and an incomplete response to ursodeoxycholic acid. Hepatol. Commun..

[9] Bowlus C.L., Yang G.X., Liu C.H., Johnson C.R., Dhaliwal S.S., Frank D., Levy C., Peters M.G., Vierling J.M., Gershwin M.E. (2019). Therapeutic trials of biologics in primary biliary cholangitis: An open label study of abatacept and review of the literature. J. Autoimmun..

[10] Zhao J., Zhao S., Zhou G., Liang L., Guo X., Mao P., Zhou X., Wang H., Nan Y., Xu D. (2011). Altered biliary epithelial cell and monocyte responses to lipopolysaccharide as a TLR ligand in patients with primary biliary cirrhosis. Scand. J. Gastroenterol..

[11] Cordell H.J., Han Y., Mells G.F., Li Y., Hirschfield G.M., Greene C.S., Xie G., Juran B.D., Zhu D., Qian D.C. (2015). International genome-wide meta-analysis identifies new primary biliary cirrhosis risk loci and targetable pathogenic pathways. Nat. Commun..

[12] Carbone M., Lleo A., Sandford R.N., Invernizzi P. (2014). Implications of genome-wide association studies in novel therapeutics in primary biliary cirrhosis. Eur. J. Immunol..

[13] Mataki N., Kikuchi K., Kawai T., Higashiyama M., Okada Y., Kurihara C., Hokari R., Kawaguchi A., Nagao S., Kondo T. (2007). Expression of PD-1, PD-L1, and PD-L2 in the liver in autoimmune liver diseases. Am. J. Gastroenterol..

[14] Nagano T., Yamamoto K., Matsumoto S., Okamoto R., Tagashira M., Ibuki N., Matsumura S., Yabushita K., Okano N., Tsuji T. (1999). Cytokine profile in the liver of primary biliary cirrhosis. J. Clin. Immunol..

[15] Gordon S.C., Trudeau S., Regev A., Uhas J.M., Chakladar S., Pinto-Correia A., Gottlieb K., Schlichting D. (2021). Baricitinib and primary biliary cholangitis. J. Transl. Autoimmun..

[16] Riva A., Chokshi S. (2018). Immune checkpoint receptors: Homeostatic regulators of immunity. Hepatol. Int..

[17] Gu D., Ao X., Yang Y., Chen Z., Xu X. (2018). Soluble immune checkpoints in cancer: Production, function and biological significance. J. Immunother. Cancer.

[18] Riva A., Palma E., Devshi D., Corrigall D., Adams H., Heaton N., Menon K., Preziosi M., Zamalloa A., Miquel R. (2021). Soluble TIM3 and Its Ligands Galectin-9 and CEACAM1 Are in Disequilibrium During Alcohol-Related Liver Disease and Promote Impairment of Anti-bacterial Immunity. Front. Physiol..

[19] Fadriquela A., Kim C.S., Lee K.J., Kang S.H., Kim M.Y., Lee J.H. (2021). Soluble type immune checkpoint regulators using multiplex luminex immunoassay in chronic hepatitis B patients. J. Clin. Pathol..

[20] Fadriquela A., Kim C.S., Lee K.J., Kang S.H., Lee J.H. (2022). Characteristics of immune checkpoint regulators and potential role of soluble TIM-3 and LAG-3 in male patients with alcohol-associated liver disease. Alcohol.

[21] Li W., Xia Y., Yang J., Guo H., Sun G., Sanyal A.J., Shah V.H., Lou Y., Zheng X., Chalasani N. (2020). Immune Checkpoint Axes Are Dysregulated in Patients With Alcoholic Hepatitis. Hepatol. Commun..

[22] Li Y.M., Shi Y.Y., Li Y., Yan L., Tang J.T., Bai Y.J., Wu X.J., Dai B., Zou Y.G., Wang L.L. (2018). Soluble Tim-3 and Gal-9 are associated with renal allograft dysfunction in kidney transplant recipients: A cross-sectional study. Int. Immunopharmacol..

[23] Javadzadeh S.M., Tehrani M., Keykhosravi M., Mohammadian-Amiri R., Amjadi O., Hafezi N., Zaboli E., Montazeriun M., Ajami A. (2022). Can we consider soluble herpes virus entry mediator (sHVEM) as a tumor marker?. Caspian J. Intern. Med..

[24] Fujimura T., Sato Y., Tanita K., Kambayashi Y., Otsuka A., Fujisawa Y., Yoshino K., Matsushita S., Funakoshi T., Hata H. (2018). Serum Level of Soluble CD163 May Be a Predictive Marker of the Effectiveness of Nivolumab in Patients With Advanced Cutaneous Melanoma. Front. Oncol..

[25] Sanmamed M.F., Perez-Gracia J.L., Schalper K.A., Fusco J.P., Gonzalez A., Rodriguez-Ruiz M.E., Oñate C., Perez G., Alfaro C., Martín-Algarra S. (2017). Changes in serum interleukin-8 (IL-8) levels reflect and predict response to anti-PD-1 treatment in melanoma and non-small-cell lung cancer patients. Ann. Oncol..

[26] Chen W., Wei Y., Xiong A., Li Y., Guan H., Wang Q., Miao Q., Bian Z., Xiao X., Lian M. (2020). Comprehensive Analysis of Serum and Fecal Bile Acid Profiles and Interaction with Gut Microbiota in Primary Biliary Cholangitis. Clin. Rev. Allergy Immunol..

[27] Tang R., Wei Y., Li Y., Chen W., Chen H., Wang Q., Yang F., Miao Q., Xiao X., Zhang H. (2018). Gut microbial profile is altered in primary biliary cholangitis and partially restored after UDCA therapy. Gut.

[28] Murray M.J., Gonze M.D., Nowak L.R., Cobb C.F. (1994). Serum D(-)-lactate levels as an aid to diagnosing acute intestinal ischemia. Am. J. Surg..

[29] Riva A., Gray E.H., Azarian S., Zamalloa A., McPhail M.J.W., Vincent R.P., Williams R., Chokshi S., Patel V.C., Edwards L.A. (2020). Faecal cytokine profiling as a marker of intestinal inflammation in acutely decompensated cirrhosis. JHEP Rep..

[30] Riva A., Patel V., Kurioka A., Jeffery H.C., Wright G., Tarff S., Shawcross D., Ryan J.M., Evans A., Azarian S. (2018). Mucosa-associated invariant T cells link intestinal immunity with antibacterial immune defects in alcoholic liver disease. Gut.

[31] Bragazzi M.C., Venere R., Vignone A., Alvaro D., Cardinale V. (2023). Role of the Gut-Liver Axis in the Pathobiology of Cholangiopathies: Basic and Clinical Evidence. Int. J. Mol. Sci..

[32] Zanoni I., Granucci F., Broggi A. (2017). Interferon (IFN)-lambda Takes the Helm: Immunomodulatory Roles of Type III IFNs. Front. Immunol..

[33] Broggi A., Tan Y., Granucci F., Zanoni I. (2017). IFN-lambda suppresses intestinal inflammation by non-translational regulation of neutrophil function. Nat. Immunol..

[34] Sommereyns C., Paul S., Staeheli P., Michiels T. (2008). IFN-lambda (IFN-lambda) is expressed in a tissue-dependent fashion and primarily acts on epithelial cells in vivo. PLoS Pathog..

[35] Ostrowski J., Goryca K., Lazowska I., Rogowska A., Paziewska A., Dabrowska M., Ambrozkiewicz F., Karczmarski J., Balabas A., Kluska A. (2019). Common functional alterations identified in blood transcriptome of autoimmune cholestatic liver and inflammatory bowel diseases. Sci. Rep..

[36] Nakagawa R., Muroyama R., Saeki C., Goto K., Kaise Y., Koike K., Nakano M., Matsubara Y., Takano K., Ito S. (2017). miR-425 regulates inflammatory cytokine production in CD4(+) T cells via N-Ras upregulation in primary biliary cholangitis. J. Hepatol..

[37] Ravichandran G., Neumann K., Berkhout L.K., Weidemann S., Langeneckert A.E., Schwinge D., Poch T., Huber S., Schiller B., Hess L.U. (2019). Interferon-γ-dependent immune responses contribute to the pathogenesis of sclerosing cholangitis in mice. J. Hepatol..

[38] Cordell H.J., Fryett J.J., Ueno K., Darlay R., Aiba Y., Hitomi Y., Kawashima M., Nishida N., Khor S.S., Gervais O. (2021). An international genome-wide meta-analysis of primary biliary cholangitis: Novel risk loci and candidate drugs. J. Hepatol..

[39] Zickert A., Oke V., Parodis I., Svenungsson E., Sundström Y., Gunnarsson I. (2016). Interferon (IFN)-λ is a potential mediator in lupus nephritis. Lupus Sci. Med..

[40] Amezcua-Guerra L.M., Márquez-Velasco R., Chávez-Rueda A.K., Castillo-Martínez D., Massó F., Páez A., Colín-Fuentes J., Bojalil R. (2017). Type III Interferons in Systemic Lupus Erythematosus: Association Between Interferon λ3, Disease Activity, and Anti-Ro/SSA Antibodies. J. Clin. Rheumatol..

[41] Eslam M., Leung R., Romero-Gomez M., Mangia A., Irving W.L., Sheridan D., Spengler U., Mollison L., Cheng W., Bugianesi E. (2014). IFNL3 polymorphisms predict response to therapy in chronic hepatitis C genotype 2/3 infection. J. Hepatol..

[42] Suppiah V., Moldovan M., Ahlenstiel G., Berg T., Weltman M., Abate M.L., Bassendine M., Spengler U., Dore G.J., Powell E. (2009). IL28B is associated with response to chronic hepatitis C interferon-alpha and ribavirin therapy. Nat. Genet..

[43] Tanaka Y., Nishida N., Sugiyama M., Kurosaki M., Matsuura K., Sakamoto N., Nakagawa M., Korenaga M., Hino K., Hige S. (2009). Genome-wide association of IL28B with response to pegylated interferon-alpha and ribavirin therapy for chronic hepatitis C. Nat. Genet..

[44] Prokunina-Olsson L., Muchmore B., Tang W., Pfeiffer R.M., Park H., Dickensheets H., Hergott D., Porter-Gill P., Mumy A., Kohaar I. (2013). A variant upstream of IFNL3 (IL28B) creating a new interferon gene IFNL4 is associated with impaired clearance of hepatitis C virus. Nat. Genet..

[45] Chen J.Y., Wang C.M., Chen T.D., Jan Wu Y.J., Lin J.C., Lu L.Y., Wu J. (2018). Interferon-λ3/4 genetic variants and interferon-λ3 serum levels are biomarkers of lupus nephritis and disease activity in Taiwanese. Arthritis Res. Ther..

[46] Suzuki T., Iwamoto N., Tsuzuki S., Kakumoto Y., Suzuki M., Ashida S., Oshiro Y., Nemoto T., Kanda K., Okuhama A. (2022). Interferon lambda 3 in the early phase of coronavirus disease-19 can predict oxygen requirement. Eur. J. Clin. Investig..

[47] Mendoza J.L., Schneider W.M., Hoffmann H.H., Vercauteren K., Jude K.M., Xiong A., Moraga I., Horton T.M., Glenn J.S., de Jong Y.P. (2017). The IFN-λ-IFN-λR1-IL-10Rβ Complex Reveals Structural Features Underlying Type III IFN Functional Plasticity. Immunity.

[48] Steinberg M.W., Cheung T.C., Ware C.F. (2011). The signaling networks of the herpesvirus entry mediator (TNFRSF14) in immune regulation. Immunol. Rev..

[49] Jung H.W., La S.J., Kim J.Y., Heo S.K., Kim J.Y., Wang S., Kim K.K., Lee K.M., Cho H.R., Lee H.W. (2003). High levels of soluble herpes virus entry mediator in sera of patients with allergic and autoimmune diseases. Exp. Mol. Med..

[50] Ware C.F., Šedý J.R. (2011). TNF Superfamily Networks: Bidirectional and interference pathways of the herpesvirus entry mediator (TNFSF14). Curr. Opin. Immunol..

[51] Heo S.-K., Ju S.-A., Kim G.Y., Park S.-M., Back S.H., Park N.-H., Min Y.J., An W.G., Nguyen T.-H.T., Kim S.-M. (2012). The presence of high level soluble herpes virus entry mediator in sera of gastric cancer patients. Exp. Mol. Med..

[52] Meng Q., Zaidi A.K., Sedy J., Bensussan A., Popkin D.L. (2019). Soluble Fc-Disabled Herpes Virus Entry Mediator Augments Activation and Cytotoxicity of NK Cells by Promoting Cross-Talk between NK Cells and Monocytes. J. Immunol..

[53] Zhao Q., Zhang G.L., Zhu X., Su D., Huang Z.L., Hu Z.X., Peng L. (2017). The paradoxical changes of membrane and soluble herpes virus entry mediator in hepatocellular carcinoma patients. J. Gastroenterol. Hepatol..

[54] Gao X., Wang X., Guan Y., Wang L., Gao Y., Niu J. (2023). Soluble immune checkpoints are elevated in patients with primary biliary cholangitis. Eur. J. Med. Res..

[55] Zhang S., Tao X., Wang L., Chen H., Zhao L., Sun J., Bian S., Chen Z., Shao T., Yang Y. (2022). Downregulation of Programmed Death-1 Pathway Promoting CD8 + T Cell Cytotoxicity in Primary Biliary Cholangitis. Dig. Dis. Sci..

[56] Schultheiß C., Steinmann S., Willscher E., Paschold L., Lohse A.W., Binder M. (2023). Immune signatures in variant syndromes of primary biliary cholangitis and autoimmune hepatitis. Hepatol. Commun..

[57] Di Leo V., Venturi C., Baragiotta A., Martines D., Floreani A. (2003). Gastroduodenal and intestinal permeability in primary biliary cirrhosis. Eur. J. Gastroenterol. Hepatol..

[58] Beaurepaire C., Smyth D., McKay D.M. (2009). Interferon-gamma regulation of intestinal epithelial permeability. J. Interferon Cytokine Res..

[59] Capaldo C.T., Nusrat A. (2009). Cytokine regulation of tight junctions. Biochim. Biophys. Acta.

[60] Vega-Magaña N., Delgado-Rizo V., García-Benavides L., Del Toro-Arreola S., Segura-Ortega J., Morales A., Zepeda-Nuño J.S., Escarra-Senmarti M., Gutiérrez-Franco J., Haramati J. (2018). Bacterial Translocation Is Linked to Increased Intestinal IFN-γ, IL-4, IL-17, and mucin-2 in Cholestatic Rats. Ann. Hepatol..

[61] Hao H., Cao L., Jiang C., Che Y., Zhang S., Takahashi S., Wang G., Gonzalez F.J. (2017). Farnesoid X Receptor Regulation of the NLRP3 Inflammasome Underlies Cholestasis-Associated Sepsis. Cell Metab..

[62] Haruta I., Kikuchi K., Hashimoto E., Nakamura M., Miyakawa H., Hirota K., Shibata N., Kato H., Arimura Y., Kato Y. (2010). Long-term bacterial exposure can trigger nonsuppurative destructive cholangitis associated with multifocal epithelial inflammation. Lab. Investig..

[63] Ma H.D., Zhao Z.B., Ma W.T., Liu Q.Z., Gao C.Y., Li L., Wang J., Tsuneyama K., Liu B., Zhang W. (2018). Gut microbiota translocation promotes autoimmune cholangitis. J. Autoimmun..

[64] Liu Y., Chen K., Li F., Gu Z., Liu Q., He L., Shao T., Song Q., Zhu F., Zhang L. (2020). Probiotic Lactobacillus rhamnosus GG Prevents Liver Fibrosis Through Inhibiting Hepatic Bile Acid Synthesis and Enhancing Bile Acid Excretion in Mice. Hepatology.

[65] Riva A. (2023). Editorial: Soluble immune checkpoints: Novel physiological immunomodulators. Front. Immunol..

[66] Prokunina-Olsson L. (2019). Genetics of the Human Interferon Lambda Region. J. Interferon Cytokine Res..

[67] Andreakos E., Zanoni I., Galani I.E. (2019). Lambda interferons come to light: Dual function cytokines mediating antiviral immunity and damage control. Curr. Opin. Immunol..

[68] Sharma L., Riva A. (2020). Intestinal Barrier Function in Health and Disease-Any role of SARS-CoV-2?. Microorganisms.

