# Supplementary material for: Soluble Herpes Virus Entry Mediator and Type II/III Interferons Are Upregulated in Primary Biliary Cholangitis
Source: Int J Mol Sci. 2025 Jan 13;26(2):605. doi: 10.3390/ijms26020605 (PMC11765339; doi:10.3390/ijms26020605)
Supplement: Supplementary file 1 [file ijms-26-00605-s001.zip › ijms-3403408-supplementary.pdf]

Article

Soluble Herpes Virus Entry Mediator and Type II/III Interferons Are Upregulated in Primary Biliary Cholangitis

Yooyun Chung [1,2,†], Hio Lam Phoebe Tsou [1,†], Michael A. Heneghan [1,2], Shilpa Chokshi [1,3,‡] and Antonio Riva [1,*,‡]

[1] The Roger Williams Institute of Liver Studies, School of Immunology and Microbial Sciences, Faculty of Life Sciences and Medicine, King's College London & Foundation for Liver Research, London SE5 9NT, UK;
[2] King's College Hospital, London SE5 9RS, UK
[3] Peninsula Medical School, Faculty of Health, University of Plymouth, Plymouth PL4 8AA, UK
* Correspondence: a.riva@researchinliver.org.uk
† Joint first authors.
‡ Joint senior authors.

Supplementary figure/table count: 2/1

Supplementary figure S1. Soluble checkpoint expression in different stages of PBC. All sol-CRs were significantly higher in end stage PBC compared to non-cirrhosis (NC). There were no differences in sol-CR levels between non-cirrhosis and early cirrhosis (EC). KWp, Kruskal-Wallis p-value. Boxplots (median, IQR, + all-value whiskers) ordered by decreasing group-wide statistical significance.

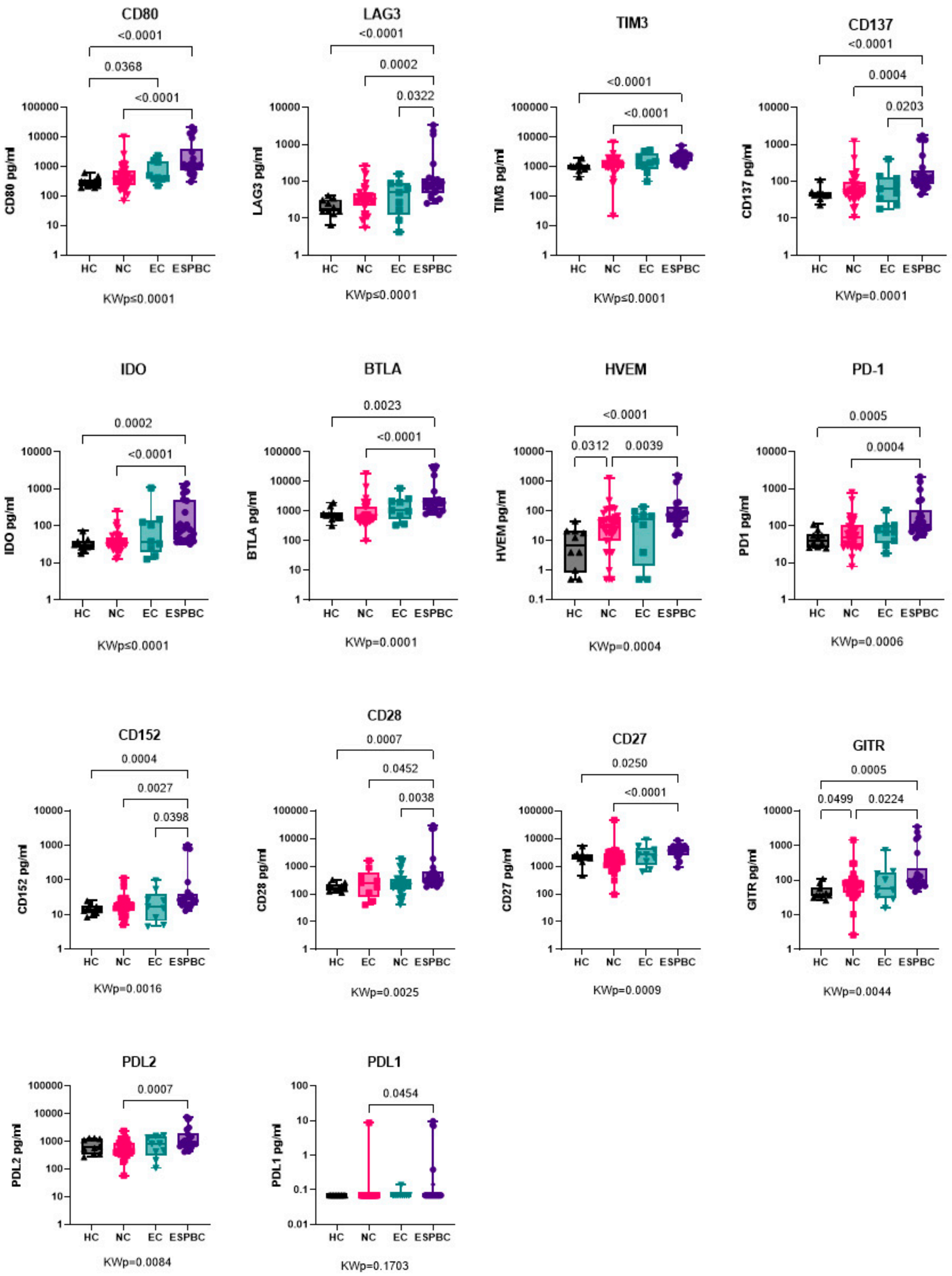

Supplementary figure S2. Boxplot representation of D-lactate in PBC according to stage of disease. Mann-Whitney p-values; Boxplots (median, IQR, ± all-value whiskers).

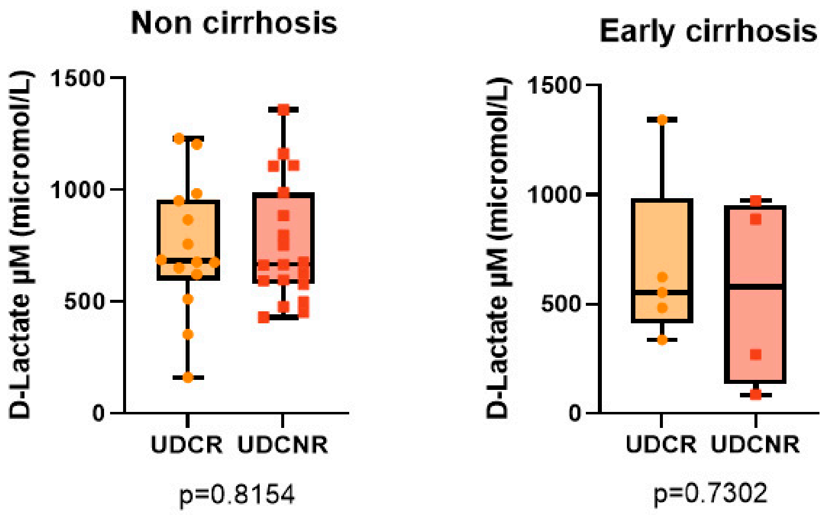

Supplementary table S1. Median (Q1 – Q3) values of soluble immune checkpoints and cytokines according to treatment response and stage of PBC (pg/mL).

		HC	UDCR	UDCNR	Non cirrhosis	Early cirrhosis	ESPBC
**Sol-CR**	**BTLA**	714.50 (586.14 – 928.97)	680.77 (523.60 – 1468.93)	584.79 (426.58 – 2182.73)	605.34 (483.06 – 1448.77)	1051.96 (506.99 – 2365.92)	1896.71 (1071.52 – 2844.46)
	**CD27**	2108.63 (1757.92 – 2722.70)	1694.34 (1145.51 – 2958.01)	1870.68 (1083.93 – 3388.44)	1733.80 (1127.20 – 2884.03)	2624.22 (1088.93 – 4602.57)	3908.41 (2471.72 – 5345.64)
	**CD28**	164.06 (136.46 – 230.67)	207.97 (101.62 – 355.63)	283.79 (179.06 – 394.46)	221.31 (149.97 – 339.63)	254.10 (78.70 – 602.56)	362.24 (267.30 – 672.98)
	**CD80**	274.79 (211.84 – 344.35)	376.70 (237.68 – 609.54)	401.79 (264.24 – 1312.20)	348.34 (223.36 – 727.78)	405.51 (376.70 – 1499.68)	1101.54 (756.83 – 4073.80)
	**CD137**	43.65 (39.54 – 51.29)	54.20 (34.51 – 90.16)	54.58 (46.03 – 124.17)	53.09 (43.15 – 96.16)	63.39 (27.80 – 129.72)	112.46 (82.41 – 191.87)
	**CD152**	13.65 (11.07 – 17.74)	16.14 (9.48 – 23.88)	19.45 (15.35 – 26.18)	18.62 (12.36 – 22.65)	16.98 (6.50 – 38.64)	26.92 (18.62 – 38.28)
	**GITR**	36.22 (34.83 – 62.66)	68.71 (37.50 – 102.33)	80.35 (50.47 – 118.03)	72.61 (43.95 – 91.83)	57.02 (30.97 – 168.27)	93.33 (67.45 – 213.80)
	**HVEM**	7.06 (0.82 – 20.89)	31.70 (10.91 – 65.01)	38.46 (3.97 – 87.70)	29.11 (9.73 – 64.27)	53.21 (1.39 – 85.11)	74.30 (40.36 – 137.09)
	**IDO**	29.44 (25.06 – 38.55)	32.81 (24.60 – 53.95)	33.73 (27.10 – 57.81)	31.92 (27.54 – 47.97)	37.50 (19.28 – 138.36)	80.35 (43.15 – 489.78)
	**LAG3**	17.38 (14.86 – 32.28)	28.44 (13.30 – 41.69)	40.74 (28.25 – 76.38)	33.27 (22.54 – 46.13)	49.89 (12.53 – 88.92)	81.28 (49.32 – 115.88)
	**PD-1**	39.99 (26.30 – 59.43)	48.87 (31.70 – 82.79)	64.42 (25.53 – 116.14)	49.32 (27.73 – 101.86)	67.30 (33.50 – 101.62)	104.71 (75.34 – 257.63)
	**PD-L1**	0.07 (0.07 – 0.07)	0.07 (0.07 – 0.07)	0.07 (0.07 – 0.07)	0.07 (0.07 – 0.07)	0.07 (0.07 – 0.07)	0.07 (0.07 – 0.07)
	**PD-L2**	609.54 (344.35 – 1253.14)	485.29 (358.10 – 818.46)	566.24 (368.98 – 1047.13)	497.74 (367.28 – 841.40)	824.14 (299.23 – 1468.93)	864.97 (663.74 – 2023.02)
	**TIM3**	1000.00 (803.53 – 1129.80)	1137.63 (893.31 – 1555.97)	1285.29 (928.97 – 1976.97)	1156.11 (899.50 – 1520.55)	1327.39 (792.50 – 2735.27)	2301.44 (1520.55 – 2576.32)
**Cytokines**	**IL-1 alpha**	1.01 (1.01 – 1.01)	1.01 (1.01 – 1.01)	1.01 (1.01 – 1.01)	1.01 (1.01 – 1.01)	1.01 (1.01 – 1.01)	1.01 (1.01 – 1.01)
	**IL-1 beta**	5.21 (5.21 – 5.21)	5.21 (5.21 – 5.21)	5.21 (5.21 – 5.21)	5.21 (5.21 – 5.21)	5.21 (5.21 – 7.36)	5.21 (5.21 – 5.21)
	**IL-1ra**	240.99 (214.78 – 331.89)	479.73 (309.03 – 972.75)	361.41 (277.97 – 635.33)	450.82 (300.61 – 805.38)	307.61 (285.76 – 1207.81)	599.79 (351.56 – 1004.62)
	**IL-2**	5.66 (5.66 – 5.66)	5.66 (5.66 – 5.66)	5.66 (5.66 – 5.66)	5.66 (5.66 – 5.66)	5.66 (5.66 – 5.66)	5.66 (5.66 – 5.66)
	**IL-4**	2.12 (2.12 – 9.10)	2.12 (2.12 – 11.91)	2.12 (2.12 – 2.52)	2.12 (2.12 – 7.94)	7.94 (2.12 – 20.37)	2.12 (2.12 – 19.36)
	**IL-5**	0.79 (0.79 – 0.79)	0.79 (0.79 – 0.79)	0.79 (0.79 – 0.79)	0.79 (0.79 – 0.79)	0.79 (0.79 – 0.79)	0.79 (0.79 – 0.79)
	**IL-6**	0.06 (0.06 – 0.06)	0.06 (0.06 – 1.12)	0.06 (0.06 – 0.35)	0.06 (0.06 – 0.71)	0.06 (0.06 – 1.71)	2.80 (0.40 – 7.96)
	**IL-7**	0.27 (0.27 – 0.27)	4.47 (0.38 – 7.73)	1.98 (0.27 – 7.85)	3.01 (0.27 – 7.60)	2.04 (0.53 – 8.61)	0.54 (0.27 – 3.49)
	**IL-8**	0.25 (0.25 – 0.30)	5.43 (2.51 – 10.16)	4.49 (1.04 – 9.14)	4.98 (2.84 – 9.82)	3.65 (0.58 – 26.61)	30.76 (8.77 – 59.43)
	**IL-10**	0.69 (0.69 – 0.69)	0.69 (0.69 – 0.69)	0.69 (0.69 – 0.69)	0.69 (0.69 – 0.69)	0.69 (0.69 – 0.69)	0.69 (0.69 – 0.69)
	**IL-12p70**	16.87 (16.87 – 16.87)	16.87 (16.87 – 16.87)	16.87 (16.87 – 16.87)	16.87 (16.87 – 16.87)	16.87 (16.87 – 16.87)	16.87 (16.87 – 16.87)
	**IL-13**	119.67 (119.67 – 119.67)	119.67 (119.67 – 119.67)	119.67 (119.67 – 119.67)	119.67 (119.67 – 119.67)	119.67 (119.67 – 119.67)	119.67 (119.67 – 119.67)
	**IL-15**	0.94 (0.94 – 0.94)	0.94 (0.94 – 0.94)	0.94 (0.94 – 0.94)	0.94 (0.94 – 0.94)	0.94 (0.94 – 0.94)	0.94 (0.94 – 0.94)
	**IL-17**	0.08 (0.08 – 0.08)	0.08 (0.08 – 0.08)	0.08 (0.08 – 0.08)	0.08 (0.08 – 0.08)	0.08 (0.08 – 0.08)	0.08 (0.08 – 0.08)
	**IL-18**	414.95 (313.33 – 557.19)	693.43 (484.17 – 1137.63)	486.41 (224.39 – 741.31)	683.91 (416.87 – 922.57)	535.80 (316.23 – 916.22)	859.01 (488.65 – 1923.09)
	**IL-23**	5.62 (5.62 – 8.38)	5.62 (5.62 – 22.08)	5.62 (5.62 – 8.38)	5.62 (5.62 – 11.25)	5.62 (5.62 – 35.24)	26.55 (5.62 – 88.72)
	**IL-33**	4.20 (4.20 – 28.51)	4.20 (4.20 – 4.20)	4.20 (4.20 – 59.16)	4.20 (4.20 – 42.76)	4.20 (4.20 – 4.20)	4.20 (4.20 – 189.67)
	**CCL2**	59.57 (42.56 – 85.51)	156.31 (103.51 – 217.77)	139.96 (70.96 – 214.29)	148.25 (102.09 – 223.36)	129.12 (72.78 – 178.65)	117.22 (69.34 – 194.09)
	**CCL3**	9.48 (9.48 – 11.27)	9.48 (9.48 – 9.48)	9.48 (9.48 – 9.48)	9.48 (9.48 – 9.48)	9.48 (9.48 – 9.48)	9.48 (9.48 – 9.48)
	**CCL4**	18.62 (18.62 – 22.13)	26.30 (18.62 – 59.02)	18.62 (18.62 – 37.24)	18.62 (18.62 – 46.88)	18.62 (18.62 – 46.88)	74.82 (31.33 – 136.14)
	**CXCL10**	126.77 (89.54 – 162.18)	205.59 (133.97 – 297.85)	168.27 (118.58 – 438.53)	188.36 (124.45 – 327.34)	250.03 (149.28 – 568.85)	671.43 (368.98 – 2228.44)
	**GM-CSF**	0.37 (0.37 – 0.37)	0.37 (0.37 – 0.37)	0.37 (0.37 – 0.37)	0.37 (0.37 – 0.37)	0.37 (0.37 – 0.37)	0.37 (0.37 – 0.37)
	**IFN-alpha**	0.00 (0.00 – 0.20)	0.00 (0.00 – 0.61)	0.00 (0.00 – 0.00)	0.00 (0.00 – 0.49)	0.00 (0.00 – 0.02)	0.00 (0.00 – 0.00)
	**IFN-gamma**	3.47 (3.47 – 3.47)	3.47 (3.47 – 3.47)	3.47 (3.47 – 116.14)	3.47 (3.47 – 34.43)	3.47 (3.47 – 3.47)	11.25 (3.47 – 147.57)
	**IFN-lambda 2 (IL-28A)**	27.04 (27.04 – 27.04)	27.04 (27.04 – 27.04)	27.04 (27.04 – 27.04)	27.04 (27.04 – 27.04)	27.04 (27.04 – 27.04)	27.04 (27.04 – 27.04)
	**IFN-lambda 3 (IL-28B)**	2.39 (2.39 – 13.96)	3.39 (2.39 – 31.33)	2.39 (2.39 – 15.31)	2.39 (2.39 – 23.60)	2.39 (2.39 – 34.43)	54.33 (19.82 – 72.95)
	**Osteopontin**	38636.70 (25644.84 – 41975.90)	26607.25 (12882.50 – 42559.84)	26001.60 (3184.20 – 48865.24)	23227.37 (10914.40 – 48083.93)	31768.74 (12705.74 – 38815.04)	40926.07 (26424.09 – 64416.93)
	**TNF-alpha**	0.09 (0.09 – 0.33)	4.11 (1.17 – 8.30)	2.62 (0.09 – 8.09)	3.91 (1.31 – 7.28)	0.09 (0.09 – 8.83)	11.32 (6.01 – 16.83)
